# Metagenomic data of vertical distribution and abundance of bacterial diversity in the hypersaline sediments of Mad Boon-mangrove ecosystem, Bay of Bengal

**DOI:** 10.1016/j.dib.2018.12.028

**Published:** 2018-12-14

**Authors:** Balamurugan Sadaiappan, Chinnamani Prasannakumar, Kumaran Subramanian, Mahendran Subramanian

**Affiliations:** a Centre for Advanced Studies in Marine Biology, Annamalai University, India; b Centre for Marine and Coastal Studies, Madurai Kamaraj University, India

## Abstract

Bacterial diversity studies in hypersaline soil often yield novel organisms and contribute to our understanding of this extreme environment. Soil from Mad Boon is previously uncharacterized, with dense mangrove forest in one side and hypersaline soil in another side of backwater located in Southeast coast of Tamil Nadu, India. We surveyed to characterize the structure and diversity of the bacterial community. Samples were collected in a partially vegetated upland, exposed backwater sedimentation and water-logged location. In this study, we investigate the bacterial community structure using pyrosequence analysis of the V5- V9 gene region. After quality checks a total of 3919, 7298 and 7399 reads were obtained. About 42 phyla were observed, among them *Proteobacteria* were dominant phylum followed by *Acidobacteria, Firmicutes* and *Chloroflexi*. Classes including *Deltaproteobacteria* and *Gammaproteobacteria*were observed. All sequences generated in this study were submitted to NCBI SRA under the accession numbers SRR627695, SRR63011 and SRR631012.

**Specification table**

**Table:** 

**Subject area**	Biology
**More specific subject area**	Metagenomics
**Type of Data**	Table, Figures and 16S rRNA Sequence
**How data was acquired**	DNA sequence Pyrosequencing was carried in Roche 454 FLX instrument at research and Testing Laboratory (RTL) (Lubbock, TX, USA)
**Data format**	Raw data sff file
**Experimental factor**	V5-V9 regions of the bacterial 16S rRNA gene were amplified using 939F-1492R oligonucleotide primers.
**Experimental features**	The sediments samples (30 cm) were collected using a corer from Mad Boon-mangrove ecosystem of Pichavaram, Tamil Nadu, India. The eDNA was extracted from the sediments based on the salinity in the sediments and sequenced using Roche 454 FLX sequencer.
**Data source and location**	Mad Boon-mangrove ecosystem of Pichavaram, Tamil Nadu, India. 11 °25’.01 N, 79 °47’ 06.39” E.
**Data accessibility**	All sequences generated in this study are submitted to NCBI SRA under the accession numbers SRR627695, SRR63011 and SRR631012
**Related research article**	Balamurugan Sadaiappan, Chinnamani Prasannakumar, Kumaran Subramanian, Mahendran Subramanian. Metagenomics approach: Vertical distribution and abundance of bacterial diversity in the hypersaline sediments of Mad Boon-mangrove ecosystem, Bay of Bengal [Draft ongoing].

**Value of the data**

• Comparison between the microbial community from different types of hypersaline sediments and this data to identify the core community is possible.
• This data is useful for comparison of saline and freshwater bacteria communities.
• Data could aid restoration of the bacterial community near the coastal regions for the agricultural process.
• The raw sequence data would allow other researchers to do their analyses with different bioinformatics tools.

## 1 Data

**Fig. 1:**
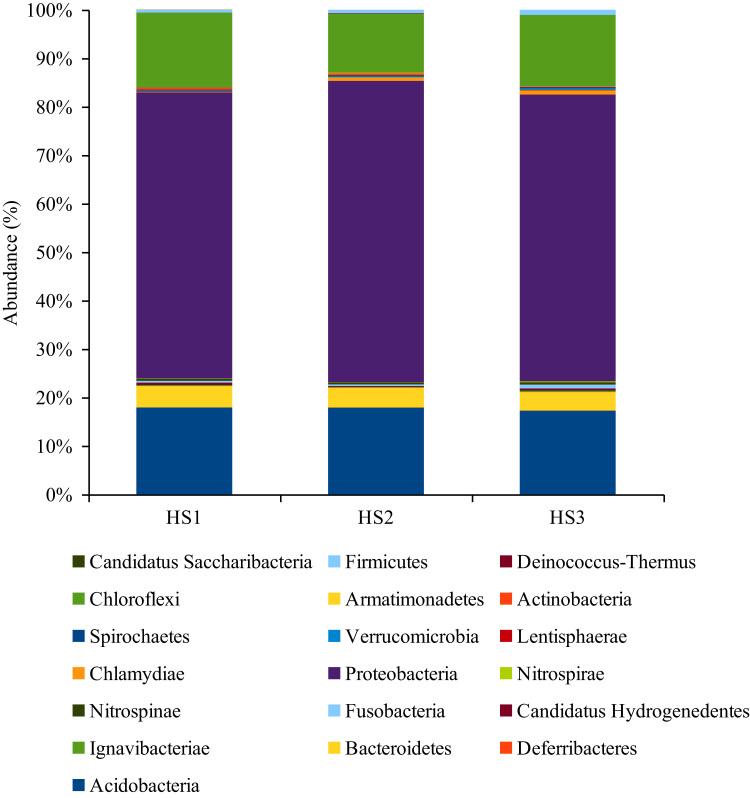
The abundance of bacterial community at the Phylum level.

**Fig. 2:**
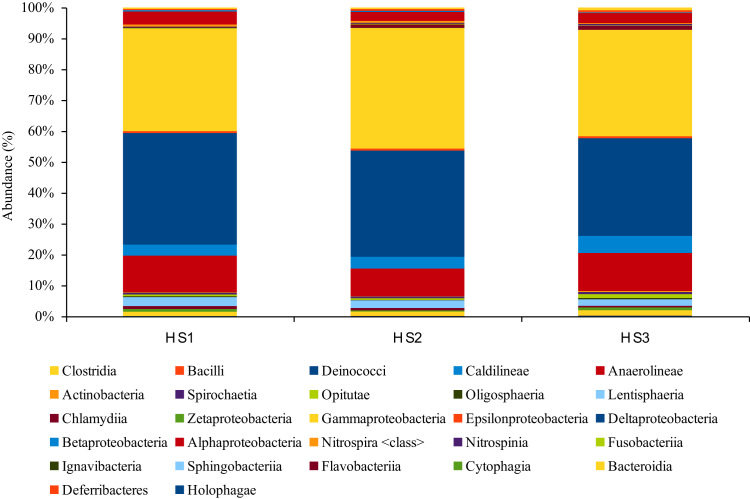
The abundance of bacterial community at the Class level.

**Fig. 3:**
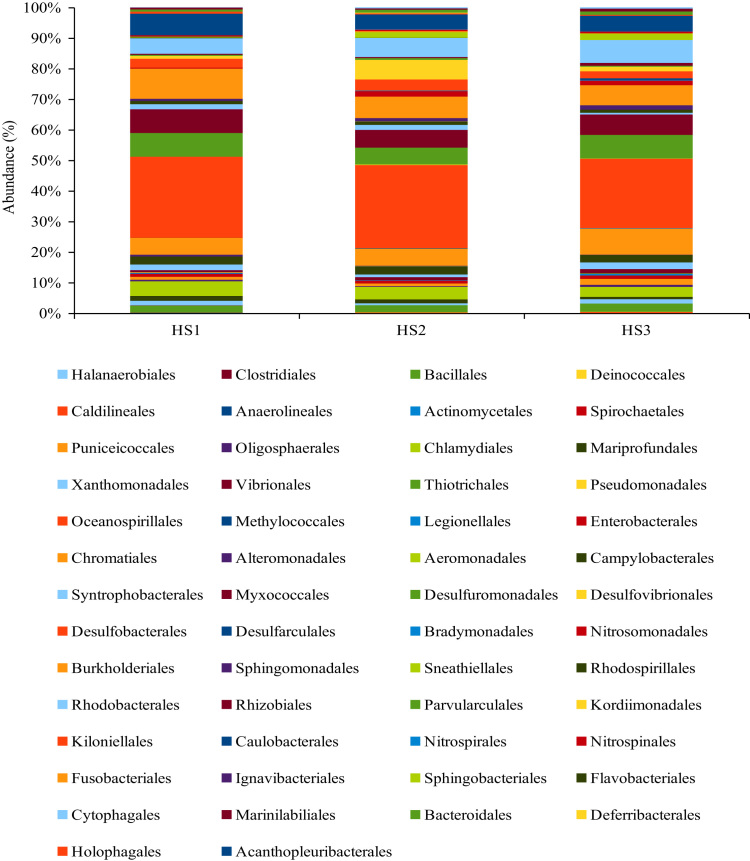
The abundance of bacteria community at the Order level.

The 16S rRNA amplicon Pyrosequencing (TEFAP, Tag-encoded amplicons Pyrosequencing) produce 5890, 13959 and 10006 sequences. After quality checks (i.e. < 250 bps lengths were removed) a total of 3919, 7298 and 7399 reads were obtained. The raw sequence can be accessed from NCBI SRA using the accession numbers SRR627695, SRR631011 and SRR631012. The phylum *Proteobacteria*was found to dominate all the three hypersaline layers followed by *Acidobacteria, Chloroflexi, Bacteriodetes* and other phyla (Fig. 1). The difference in the bacterial communities was seen even in class (Fig. 2), order (Fig. 3), family and genus level (SupplementaryData Table 1 and Supplementary Data Table 2 respectively), Diversity richness Shannon and cho1 index (Table 1).

**Table 1:** Hypersaline bacterial diversity, richness derived from multiple diversity estimators for individual sediment samples 3%, OTUs, Shannon and Chao1 index of the samples.

Vertical Location Sample ID	Sequence library	Number of	Shannon	Chao I	ACE
	size	OTUs^a^	(H’)	richness	
HS1	3919	1164	6.48703	1,436.81	1436.81
HS2	7026	1645	6.72401	1,950.66	1886.87
HS3	7377	1547	6.64978	1,843.67	1547

## 2 Experimental design, material and methods

### 2.1 Sample collection

The study was conducted in Mad Boon (Pichavaram), South India, Bay of Bengal in a natural area, located at Latitude 11 °25’.01 N; Longitude 79 °47’ 06.39” E in September 2012. The sample was collected in a single location using 10.5 cm wide and 40 cm height corer. The top 0–30 cm sediments were sampled and transferred to the lab using sterilized polyethylene bags. The sediments were separated into three bases on the salinity top 1–5 cm HS1 (110 PSU), middle 6–15 cm (85 PSU) and bottom 16–30 cm (67 PSU). Physico-chemical properties of sediments were analysed in soil testing lab, Chidambaram, Tamil Nadu (Table 2) followed by the procedure in [1].

**Table 2:** Abiotic parameters of hypersaline soil.

Physico chemical characters	Sample
Temperature (°C)	30 ± 1.2
pH	7.6 ± 0.3
Salinity (PSU)	117 ± 2.672
	Macronutrients (mg/g)
Total N	6.2 ± 1.50
P	1.92 ± 0.062
K	9.0 ± 1.98
NH_4_	5.76 ± 3.9
S0_4_	6.59 ± 1.67
Total organic C	30.0 ± 7.23
	Micronutrients and heavy metals (µg/g)
Al	135.4 ± 2.03
B	0.631 ± 0.066
Cd	0.041 ± 0.004
Co	11.59 ± 0.325
Cr	1.979 ± 0.023
Cu	0.335 ± 0.002
Fe	129.7 ± 5.06
Mg	43.13 ± 3.23
Mn	1.81 ± 0.072
Ni	184.4 ± 9.54
Pb	0.309 ± 0.008
Zn	9.756 ± 2.4

### 2.2 DNA extraction

Briefly, 1 g of sediments were taken from each layer and the DNA extraction was done by using FASTDNA SPIN Kit for soil (Qiagen, Valencia, CA, USA). DNA extraction was conducted in triplicate. The quality and concentration of extracted eDNA were estimated using 1% agarose gel and Nanodrop ND-2000 (Thermo Scientific) [2].

### 2.3 Sequencing

The quality and concentration of eDNA were estimated. Next, the corresponding eDNA were pooled together. 100 ng of eDNA from each layer, 16S rRNA specific V5-V9 primers [3] 939F (5’TTGACGGGGGCCCGCAC3’) and 1492R (5’TACCTTGTTACGACTT3’) primer were used to amplify the fragments using the titanium reagents, Hot Star Taq Plus master mix Kit (Qiagen, Valencia, CA). TEFAP procedures and sequencing were carried out at the Research and Testing Laboratory (RTL), Lubbock, TX, USA using Genome Sequencer FLX System (Roche, Nutley, N.J., USA) based on RTL protocols.

### 2.4 Data analysis

The TEFAP generated sff files (Three) were submitted to NCBI׳s Bio project, BioSample, and SRA. The sequences were quality trimmed according to the procedure in [4], and the sff files were converted into FASTA and QUAL file formats using Mothur v.1.12.0 [5]. Then the sequence was trimmed, aligned, filtered, and the chimers were removed using Mothur commands. Then, the sequences were classified against Silva Database [6]. The sequence data generated in this study can be downloaded from NCBI SRA.
