# Supplementary material for: Metagenomic data of vertical distribution and abundance of bacterial diversity in the hypersaline sediments of Mad Boon-mangrove ecosystem, Bay of Bengal
Source: Data Brief. 2018 Dec 14;22:716–21. doi: 10.1016/j.dib.2018.12.028 (PMC6329363; doi:10.1016/j.dib.2018.12.028)
Supplement: Supplementary file 2 — Supplementary material [file mmc2.pdf]

Supplementary Data Table 1: Difference in the abundance of bacteria community at the Family level in Hypersaline (HS) sediments layers	HS1	HS2	HS3
Acanthopleuribacteraceae	1	0	0
Holophagaceae	1	2	4
Marinifilaceae	1	0	0
Flammeovirgaceae	7	3	12
Cryomorphaceae	6	5	1
Flavobacteriaceae	12	10	7
Ignavibacteriaceae	6	3	8
Fusobacteriaceae	11	10	23
Nitrospinaceae	11	11	13
Nitrospiraceae	3	2	6
Caulobacteraceae	1	0	1
Kiloniellaceae	1	0	1
Kordiimonadaceae	2	0	0
Parvularculaceae	0	0	2
Aurantimonadaceae	0	0	2
Brucellaceae	0	2	3
Hyphomicrobiaceae	0	1	3
Phyllobacteriaceae	1	1	1
Rhodobiaceae	2	4	1
Xanthobacteraceae	1	0	0
Rhodobacteraceae	21	10	26
Rhodospirillaceae	17	19	9
Sneathiellaceae	0	1	0
Erythrobacteraceae	5	0	1
Alcaligenaceae	3	6	6
Burkholderiaceae	59	55	93
Comamonadaceae	0	2	1
Oxalobacteraceae	0	0	1
Thiobacillaceae	1	0	0
Bradymonadaceae	0	0	2
Desulfarculaceae	0	2	0
Desulfobacteraceae	143	153	154
Desulfobulbaceae	147	160	113
Desulfohalobiaceae	0	2	0
Desulfovibrionaceae	0	0	1
Desulfuromonadaceae	63	42	43
Geobacteraceae	0	0	1
Kofleriaceae	7	1	4
Nannocystaceae	18	11	8
Polyangiaceae	5	2	2
Sandaracinaceae	17	5	8
Syntrophaceae	0	0	1
Syntrophobacteraceae	0	0	1
Campylobacteraceae	6	2	1
Helicobacteraceae	6	10	11
Aeromonadaceae	0	1	0
Alteromonadaceae	7	9	8
Colwelliaceae	0	0	3
Idiomarinaceae	0	1	3

Family

Chromatiaceae	0	4	3
Ectothiorhodospiraceae	33	30	36
Thioalkalispiraceae	10	5	3
Enterobacteriaceae	4	21	18
Coxiellaceae	0	2	2
Methylococcaceae	0	0	7
Alcanivoracaceae	1	1	1
Halomonadaceae	6	5	3
Kangiellaceae	3	1	0
Oceanospirillaceae	13	27	19
Saccharospirillaceae	4	3	3
Moraxellaceae	7	72	15
Pseudomonadaceae	4	2	3
Piscirickettsiaceae	4	7	4
Vibrionaceae	4	4	11
Xanthomonadaceae	56	70	87
Mariprofundaceae	3	1	0
Oligosphaeraceae	1	0	0
Puniceicoccaceae	0	4	1
Spirochaetaceae	4	6	7
Anaerolineaceae	81	55	60
Caldilineaceae	8	4	4
Trueperaceae	0	1	0
Bacillaceae	0	4	6
Planococcaceae	0	3	0
Clostridiales Family XII. Incertae Sedis	1	1	4
Defluviitaleaceae	0	0	1
Peptostreptococcaceae	0	1	0
Ruminococcaceae	1	2	0
Halanaerobiaceae	1	4	5
