# Supplementary material for: Metagenomic data of vertical distribution and abundance of bacterial diversity in the hypersaline sediments of Mad Boon-mangrove ecosystem, Bay of Bengal
Source: Data Brief. 2018 Dec 14;22:716–21. doi: 10.1016/j.dib.2018.12.028 (PMC6329363; doi:10.1016/j.dib.2018.12.028)
Supplement: Supplementary file 3 — Supplementary material [file mmc3.pdf]

Supplementary Data Table 2: Difference in the abundance of bacteria community at the Genus level in Hypersaline (HS) sediments layers	HS1	HS2	HS3
Acanthopleuribacter	1	0	0
Thermoanaerobaculum	2	0	0
Marinifilum	1	0	0
Fulvivirga	0	1	2
Actibacter	1	1	0
Gramella	0	2	0
Muricauda	0	1	0
Robertkochia	1	0	2
Robiginitalea	5	1	2
Candidatus Hydrogenedens	15	6	13
Propionigenium	4	4	7
Nitrospina	11	11	13
Nitrospira	3	2	6
Brevundimonas	1	0	1
Kiloniella	1	0	1
Kordiimonas	2	0	0
Martelella	0	0	2
Anderseniella	0	1	1
Bauldia	1	0	0
Pseudolabrys	1	0	0
Roseovarius	0	0	1
Tropicimonas	0	0	1
Magnetovibrio	0	0	1
Pelagibius	14	17	7
Thalassospira	0	2	0
Sneathiella	0	1	0
Altererythrobacter	4	0	1
Achromobacter	1	5	3
Burkholderia	57	55	93
Pelomonas	0	0	1
Thiobacillus	1	0	0
Bradymonas	0	0	2
Desulfatiglans	9	10	7
Desulfatitalea	4	5	3
Desulfonema	1	0	1
Desulfosalsimonas	3	3	1
Desulfosarcina	6	13	4
Desulfobulbus	1	1	0
Desulfopila	0	0	5
Desulforhopalus	0	0	2
Desulfurivibrio	0	2	1
Desulfovibrio	0	0	1
Desulfuromonas	3	5	5
Malonomonas	1	2	0
Pelobacter	3	1	4
Geoalkalibacter	0	0	1
Haliangium	5	0	4
Kofleria	2	1	0

Sandaracinus	17	5	8
Desulfomonile	0	0	1
Deferrisoma	16	16	19
Arcobacter	6	2	1
Sulfurimonas	0	1	4
Alishewanella	0	2	2
Alteromonas	0	0	1
Marinobacter	2	2	2
Thalassotalea	0	0	3
Idiomarina	0	1	3
Thioalkalivibrio	3	2	2
Thiohalophilus	1	3	2
Enterobacter	0	1	2
Coxiella	0	2	0
Alcanivorax	0	1	0
Halomonas	6	4	3
Pleionea	3	1	0
Marinobacterium	2	19	2
Neptuniibacter	3	0	6
Gynuella	2	0	0
Saccharospirillum	2	3	3
Acinetobacter	7	72	14
Pseudomonas	2	1	2
Methylophaga	4	7	4
Litorivivens	0	1	0
Vibrio	4	3	9
Stenotrophomonas	53	69	87
Mariprofundus	3	1	0
Oligosphaera	1	0	0
Coraliomargarita	0	4	0
Spirochaeta	1	1	1
Litorilinea	1	2	0
Truepera	0	1	0
Bacillus	0	1	2
Halobacillus	0	3	3
Oceanobacillus	0	0	1
Solibacillus	0	2	0
Fusibacter	1	1	4
Vallitalea	0	0	1
Ercella	1	1	0
